# Applying a multidisciplinary intervention for drug adequacy in an intermediate care hospital (AMIDA-ICH): study protocol of a complex intervention trial

**DOI:** 10.3389/fmed.2026.1857969

**Published:** 2026-08-19

**Authors:** Francesco Salis, Nuria Soler-Blanco, Núria Molist-Brunet, Neus Gual-Tarrada, Aida Ribera, Antonio Cherubini, Marco Inzitari, Ana María de Andrés-Lázaro

**Affiliations:** 1 RE-FiT Barcelona Research Group, Vall d’Hebrón Institute of Research (VHIR), Barcelona, Catalunya, Spain; 2 Department of Medical Sciences and Public Health, University of Cagliari, Cagliari, Italy; 3 Parc Sanitari Pere Virgili, Barcelona, Catalunya, Spain; 4 Universitat de Vic-Universitat Central de Catalunya (UVic-UCC), Vic, Catalunya, Spain; 5 Universitat Autònoma de Barcelona, Barcelona, Catalunya, Spain; 6 Vall d’Hebrón University Hospital, Barcelona, Catalunya, Spain; 7 CIBER de Epidemiología y Salud Pública (CIBERESP), Barcelona, Catalunya, Spain; 8 Geriatria, Accettazione Geriatrica e Centro di ricerca per l'invecchiamento, IRCCS INRCA, Ancona, Italy; 9 Department of Clinical and Molecular Sciences, Università Politecnica delle Marche, Ancona, Italy; 10 Faculty of Health Sciences, Universitat Oberta de Catalunya (UOC), Barcelona, Catalunya, Spain

**Keywords:** complex interventions, geriatrics, implementation, integrated care, person-centred prescription

## Abstract

**Introduction:**

Person-centred prescription (PCP) is a medication revision based on patients’ frailty status and diagnostic- and drug-centred criteria. This pragmatic trial aims to implement and evaluate a multidimensional PCP intervention to improve pharmacological treatment adequacy in older adults admitted to an intermediate care hospital (ICH), following a mixed-method approach.

**Description:**

The “Applying a Multidisciplinary Intervention for Drug Adequacy-ICH” (AMIDA-ICH) study follows a hybrid design. Five hospital wards will enter the intervention phase following a stepped–wedge randomized clinical trial design. Subjects belonging to the control phase will receive usual care; those belonging to the intervention phase will undergo a multidisciplinary PCP. We will evaluate the intervention’s impact on reducing medications and improving treatment adequacy, patient-reported outcome measures, and health outcomes, such as adverse events, hospital admissions, and mortality. Finally, the qualitative part will involve professionals, patients, and carers with the aim of identifying barriers and enablers to implement the intervention.

**Discussion:**

This study is designed to improve therapeutic adequacy and quality of care in older adults through a PCP approach. It focuses on outcomes like efficacy and safety, while also assessing the real-world applicability of the intervention through implementation outcomes.

**Clinical trial registration:**

https://clinicaltrials.gov/study/NCT07015112, NCT07015112.

## Highlights

- Hybrid mixed-methods design combining a stepped-wedge cluster randomized trial with qualitative evaluation
- Multidisciplinary person-centred prescription model, based on frailty
- Evaluation of medication appropriateness and key clinical outcomes, including adverse events, readmissions and mortality
- Single-center study, which may limit the generalisability of findings.

## Introduction

The current demographic landscape is characterized by increased life expectancy (1), driven by a genetic background on which lifestyle and environment have significant weight (1). Nonetheless, it does not appear to be mirrored by an equally significant increase in healthy life expectancy (1, 2).

The concept of frailty has therefore become increasingly relevant in the literature as well as in clinical practice, as it highlights the complex interplay of factors called into question in driving treatment intensity and goals of care in the older population.

Frailty has not a universally shared definition, although it is commonly described as a dynamic, age-related, multidimensional “biopsychosocial” syndrome, characterized by declined functioning in different geriatric domains, and increased vulnerability to stressors (3, 4). It is also defined as a state of poor health deriving from the accumulation of deficits, rather than a syndrome (5). Whatever the definition, it represents a significant public health concern, making patients more vulnerable to complications, disability and a higher risk of institutionalization (4). A systematic review of 240 population-based studies, including almost 1,800,000 community-dwelling people over 62 countries, aged 50 years and older, showed that the prevalence of frailty as a “physical syndrome” is around 12%, while frailty as a deficit accumulation is around 24% (6). Frailty also seems to depend on the number of chronic diseases (7), and excluding patients with multiple diseases and higher degrees of frailty from clinical trials surely represents a limitation of the scientific literature and the clinical practice as well.

Moreover, a significant comorbidity burden usually involves a just as significant medication burden. The term “polypharmacy” arises in this context, being recognized as a geriatric syndrome. As for frailty, polypharmacy does not have a commonly shared definition. It usually refers to the chronic need for 5 or more medications (8), but some other definitions have been proposed in the literature. Some works also consider the duration of the treatment or qualitative criteria (8). This suggests that, regardless of the mere quantity, certain medications may be inadequate and potentially inappropriate in certain specific population groups. A systematic review of 106 observational studies on young and older adults showed a pooled polypharmacy prevalence of 37%, regardless of the definition (9). This review included inpatients, as well as outpatients and community-dwelling people, demonstrating the association between polypharmacy and age (65 years and older), and also clinical setting – related to the level of intensity of care –, but not with geography nor gender (9). Polypharmacy and frailty appear to be mutually related. A secondary analysis of a large randomized-controlled trial conducted in US and Australia showed that people chronically taking 5 or more medications had a relative risk ratio of 1.15 for pre-frailty and 3.34 for frailty, and that polypharmacy increased the disability-free survival period loss in both pre-frail and frail people (10). Several authors suggest that the relationship between polypharmacy and frailty is bidirectional (7, 11, 12). Although most of the studies are not designed to determine this association, it seems that medications can influence frailty and are directly involved in its pathogenesis (7, 12). Moreover, this link remains also when considering frailty and potentially inappropriate medications (PIMs) (11), anticholinergic burden, and adverse drug events (ADEs) (12). Also, despite the prevalence of polypharmacy increasing worldwide, there is still a high prevalence of underprescription in the older population, which might contribute to the negative consequences of inappropriate prescription (13).

Multiple tools are available to assist clinicians with the process of medication optimization, the majority of which are explicit, that is lists of medications that should not be prescribed in older people because of an unfavorable benefit–risk profile (14), and, for some of them, lists of medication that should be prescribed because of a valid indication, such as the STOPP and START criteria (6). Although widespread and useful, they cannot fully address all the heterogeneity displayed by the older adult population. For these reasons, other strategies should be implemented. According to available evidence in older patients with polypharmacy, the medication review is a key strategy (12, 15). It is explained that the revision faces patient-, provider- and system-based barriers, and even if it may have no significant impact on mortality or ADEs, it can improve the quality of life, increase deprescription, and reduce health costs and readmissions (12, 16). In particular, an evidence-based approach such as person-centred prescription (PCP) should be recommended. It refers to a multiprofessional approach aimed at improving the quality of care. The literature offers some models, implemented in different countries and contexts, and the role of the patients is always crucial (17, 18). These models consider individual characteristics to enhance appropriate medication use, adherence, and safety, with different instruments (16, 19, 20). Generally speaking, to achieve it, in response to a “medication-oriented” approach, PCP focuses more on subjective clinical context. Moreover, in this regard, therapeutic plans should be agreed with the patients and their caregivers (21, 22).

There is growing interest in enhancing patient care through more seamless transitions and support systems. In the realm of continuity of care, intermediate care services represent transitional time-limited services which ensure continuity of care, promote recovery, independence and confidence at the interface between home and acute services (23, 24), thus shortening or avoiding unnecessary admissions to acute wards, even with debated efficacy (24).

### Problem statement

Although PCP should be considered for all older frail patients, its implementation is often challenging in certain settings due to structural limitations, a predominantly disease-oriented approach, and/or limited time. Intermediate Care Hospitals (ICHs) may offer an optimal environment for conducting patient-centred medication reviews, for several reasons. Multidisciplinary teams, including geriatricians, nurses, pharmacists, physiotherapists, occupational therapists, and social workers, among others, operate in ICH. Comprehensive geriatric assessment, enablement and rehabilitation are implemented, with a focus on optimizing recovery and independence, and, when possible, improving frailty status after an acute event. Moreover, the relatively extended stay of patients supports a more thorough and multidimensional approach to “functional recovery.” Nonetheless, despite representing an ideal environment, evidence on the patient-centred prescription process in ICH is still lacking.

Given the above-mentioned background, the implementation of an intervention based on PCP review by a multidisciplinary team (geriatrician, clinical pharmacist, nurse) might improve the adequacy of treatments and reduce the level of polypharmacy in the older population admitted to ICHs. Also, the inclusion of patient views and preferences regarding their treatment during the PCP review might help to achieve success in reducing polypharmacy, i.e., deprescribing, and improving adherence (25). The goal of the intervention is therefore to maximize benefits of pharmacological treatment, and concurrently reduce medication-related risks, such as ADEs.

### Objectives

The objective of this trial is the implementation of a multidimensional intervention addressed at improving adequacy of pharmacological treatment in older adults admitted to an ICH.

Different dimensions of the proposed intervention will be assessed in parallel. We will evaluate the impact of the intervention on reducing the number of medications per patient and polypharmacy prevalence, and on the adequacy and safety of treatments, including ADEs, unplanned readmissions, and mortality. We will also evaluate patients’ preferences and attitudes towards pharmacological treatment during the medication review process, together with barriers and enablers for the implementation of the intervention.

## Description of the methodology

### Design

This study will follow a type 2 hybrid design (26) to implement and evaluate a complex intervention, using mixed methods. In detail, we will conduct a single centre, pragmatic, stepped-wedge randomized cluster open-label trial design (27). The units of randomization will be five geriatric rehabilitation units of the ICH (5 clusters). The unit of analysis will be the patient.

**Figure 1:**
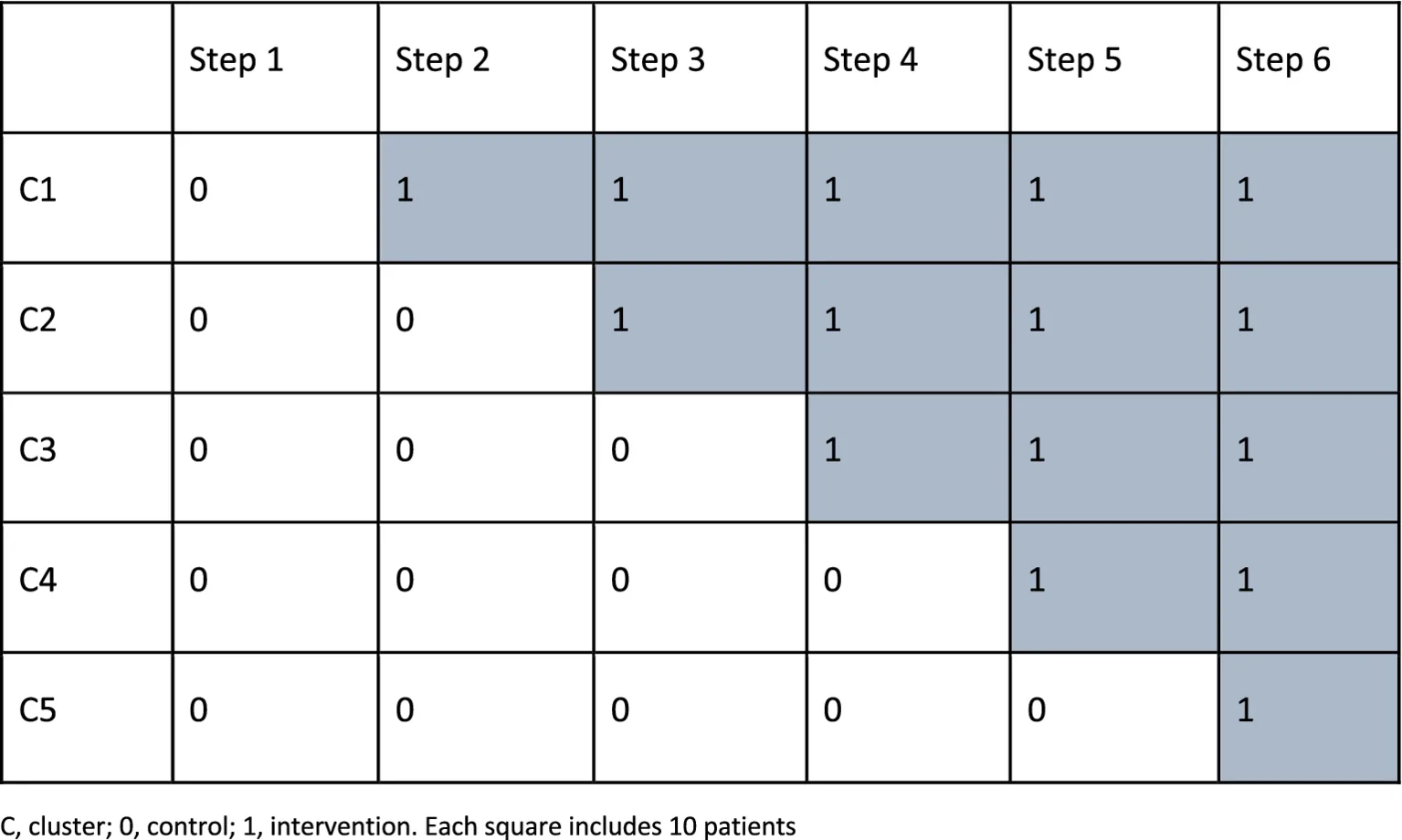
Stepped-wedge trial design. C, cluster; 0, control; 1, intervention. Each square includes 10 patients.

All clusters (hospital wards) will begin the study under the control condition. Subsequently, they will be randomly assigned to the intervention at predetermined time points in a sequential, staggered manner until all clusters receive the intervention (see Figure 1).

The computer-generated stepwise randomization of clusters will be carried out with the statistical software R with a randomization seed 184.

For the identification of barriers and enablers for the implementation, we will follow qualitative analysis combining techniques of semi-structured interviews and focal groups.

Trial registration (clinical trial number): NCT07015112 (June 10, 2025).

### Study setting

The study will be developed in an urban ICH “Parc Sanitari Pere Virgili,” Barcelona, Spain. The facility includes 360 inpatient beds, divided into geriatric rehabilitation, subacute care, palliative care, and long-term care, and approximately 40 hospital-at-home beds.

Patients will be included from the five geriatric rehabilitation wards. Most of the admitted patients usually come from a hip fracture or a stroke, or they are admitted following a stay in an acute care ward. The medical staff consists of geriatricians and general practitioners, and multidisciplinary team includes nurses, pharmacists, physiotherapists, occupational therapists, psychologists and social workers, among others. The average length of stay per patient is around 30 days.

### Inclusion and exclusion criteria

Inclusion criteria: older adults (≥65 years), admitted to geriatric rehabilitation units, with 3 or more chronic conditions requiring pharmacological therapy, able to speak Spanish and with the capacity to provide informed consent (or with a carer able to consent on their behalf), under the care of a multidisciplinary team, including geriatricians.

Exclusion criteria: inability to provide informed consent, anticipated length of hospital stay <72 h, or estimated life expectancy <3 months, homeless, patients already enrolled in a drug trial.

Participants who meet the inclusion criteria will be selected upon admission until the necessary sample size is reached.

### Intervention

**Figure 2:**
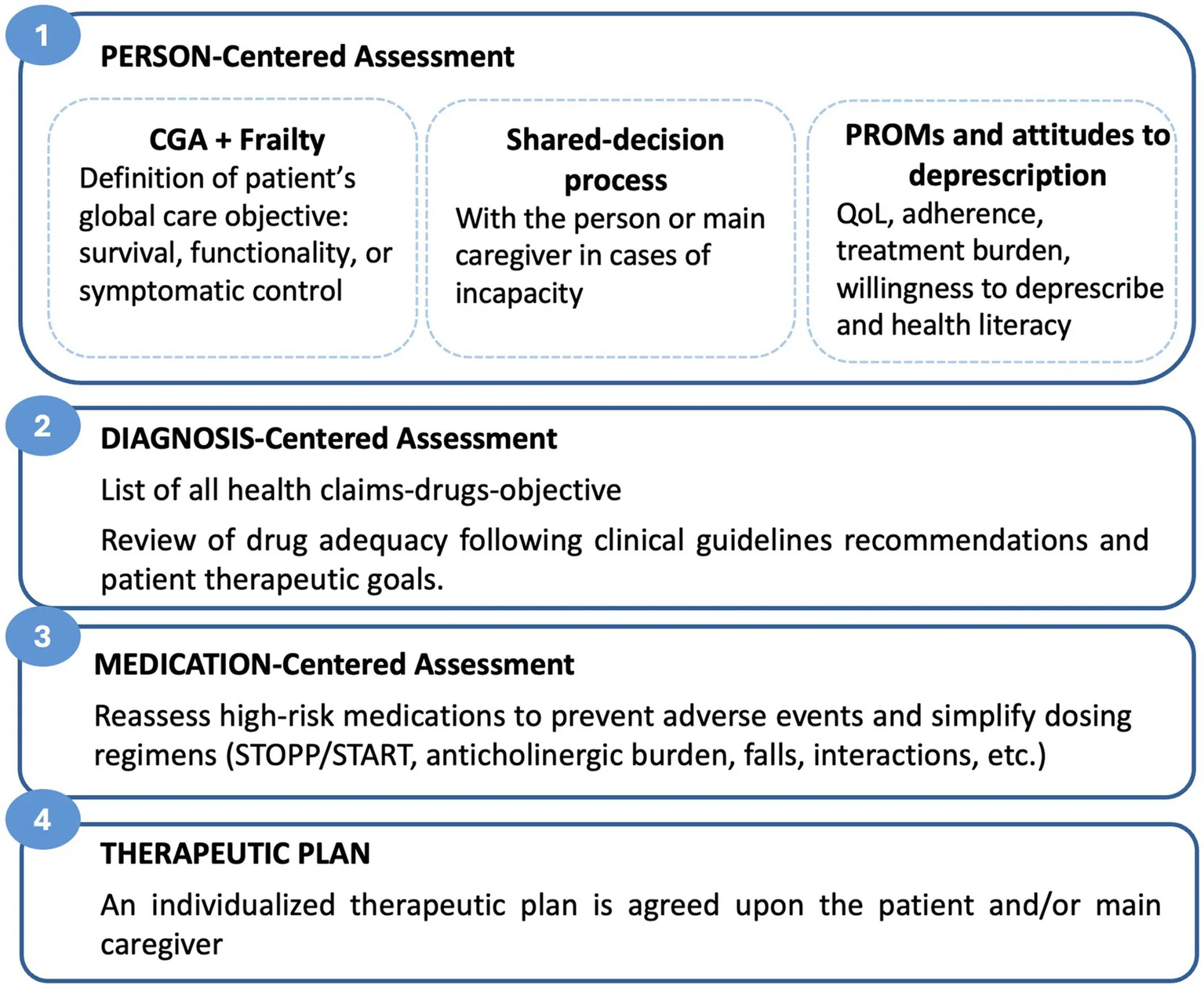
Methodology followed in the intervention phase. CGA, comprehensive geriatric assessment; PROMs, patient-reported outcome measures; QoL, quality of life.

In experimental phase, patients will undergo a multidisciplinary review (geriatrician, nurse, clinical pharmacist) of pharmacological treatment upon admission, according to a PCP methodology structured in 4 steps (28): (1a) Person-centred evaluation (person insights): person attitudes and preferences regarding treatment; patient reported outcomes (adherence, burden of treatment, willingness to deprescribe and health status self-assessment). (1b) Person-centred evaluation (clinical insights): comprehensive geriatric evaluation, and definition of therapeutic goals set by the health care team, taking into account the patient preferences. (2) Diagnostic-centred evaluation: review of specific health problems and prescribed chronic treatments considering both current clinical guidelines and the abovementioned treatment intensity. (3) Drug-centred evaluation: identification of drugs with a high iatrogenic potential, interactions, duplications, adjustments for renal/hepatic function and pharmaceutical adequacy. (4) Final therapeutic plan and implementation. Figure 2 shows the followed PCP methodology.

### Control

In the control phase, patients will undergo usual care, described as intermediate medication review, that can be considered as the standard of care (29). It is a reconciliation and treatment review by clinical pharmacists upon admission, considering clinical data, without direct contact with the patients.

### Outcomes

The primary outcome of the study is the mean number of medications per patient.

Secondary outcomes are (see also “Participant timeline and data collection”): prevalence of quantitative polypharmacy and hyperpolypharmacy, qualitative polypharmacy, PIMs, anticholinergic burden, number of underprescribed medications that have been started, ADEs and adverse drug withdrawal events (ADWEs), unplanned care utilization, and mortality. Patient-reported outcome measures (PROMs) will also be secondary outcomes for this study. They include adherence, treatment burden, perceived health status and quality of life, health literacy, and attitudes on deprescription (see Table 1). Finally, another secondary outcome will be the identification of barriers and facilitators of the implementation of the purposed methodology, both at the level of the individual person/healthcare professionals (possible cultural aspects, preferences, etc.) as well as from the system point of view (aspects related to intermediate care).

**Table 1:** Detailed collected variables.

Demographic variables	Multidimensional assessment	Medication assessment	PROMs
Age Sex Education Family support	Frailty, according to the FI-VIG (53) Dependence in activities of daily living, according to the Barthel Index (54) Dependence on medication management Morbidity, according to the age-adjusted Charlson Comorbidity Index (55) Cognitive impairment, according to the Pfeiffer test (54, 56) Delirium, according to the 4 ‘A’s Test (57) Nutritional status, according to the Mini-Nutritional Assessment – short form (58) Risk of pressure injuries, according to the Braden Scale (59) Need for palliative care, according to NECPAL (60)	Prevalence of quantitative polypharmacy and hyperpolypharmacy (number of patients taking ≥5 and ≥10 medications, respectively; long-term medications will be estimated as the sum of every regularly scheduled medication intended to be administered for a period ≥3 months, including medications with “on demand” dosage) Qualitative polypharmacy: classification of the treatments according to treatment objective (preventive – primary or secondary prevention –, curative/etiological, symptomatic) PIMs, according to the EU(7)-PIM list (61) Anticholinergic burden, according to the ACB score (62)	Adherence, according to the ARMS-e scale (63) Treatment burden, according to the Treatment Burden Questionnaire (64) Health status self-assessment, modified from the Spanish National Health Survey (65) Health-related quality of life, according to the EuroQoL-5D (66) Health literacy, according to a set of 18 health-related words, where the patient is asked to associate each word with either a key term or a distractor from a pair Attitudes on deprescription, according to rPATD questionnaire (67)

### Sample size and recruitment

Sample size was calculated using the Stata command stepped-wedge, considering 5 clusters, a size of each cluster *N* = 10 and five times of measurement after baseline, incorporating one cluster to the intervention phase at each time. Based on previous studies (21), taking a baseline mean drugs of 8.81 and expected decrease of 1.31, we considered an ICC = 0.2 (intraclass correlation). With this data, a sample size of 300 subjects will provide a power of 75%. The sample size was calculated by a statistician within the research group.

For the qualitative part, we aim to recruit and interview 15 older patients with different frailty profiles (mild, moderate, and severe frailty), from both genders. Caregivers of those patients, especially the most severely frail, and 5 geriatricians will also be invited to join the study. We estimate a total of 20–30 participants in this group. Additionally, we aim to include a minimum of 4 focal participation groups (between 5 and 10 people per group), including both patients and healthcare professionals (geriatricians, pharmacists and nurses). The selection of the group participants will be performed to obtain a balanced representation of the different profiles.

### Participant timeline and data collection

Enrolled patients will be evaluated upon admission for the variables detailed in Table 1 (baseline assessment) and undergo medication review (see “Interventions”). Since the planned intervention will be performed upon hospital admission, we expect nearly total retention. In detail, a trained clinical pharmacist and a geriatrician will perform an evaluation on demographic data, a brief multidimensional geriatric assessment, including the evaluation of the frailty, and data on quantitative/qualitative polypharmacy.

**Figure 3:**
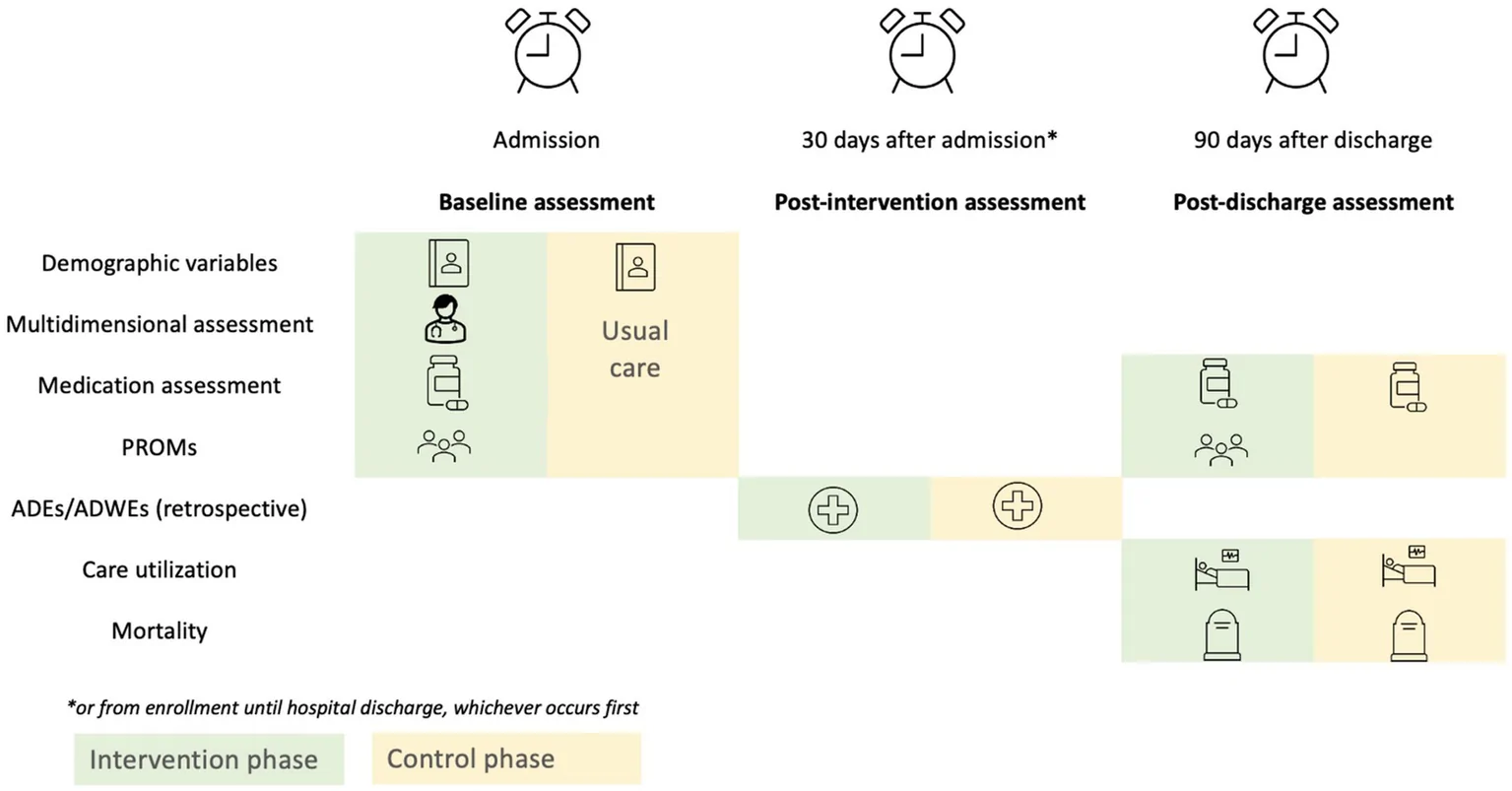
Timeline of included subjects. ADEs, adverse drug events; ADWEs, adverse drug withdrawal events; PROMs, patient-reported outcome measures.

For the intervention phase, PROMs will be assessed upon admission through an interview conducted by a trained clinical pharmacist (see Table 1). Figure 3 summarizes the timeline for the intervention phase.

In both intervention and control phase, ADEs/ADWEs will be identified by research staff based on a retrospective review of medical records from enrolment to index hospital discharge, or 30 days post recruitment, whatever comes first (post-intervention assessment). We will apply systematic trigger tools suggestive of ADEs/ADWEs occurrence (see Table 2). In detail, the final list is derived from the integration of different validated trigger tools that have been previously used in similar populations. The list was reviewed and validated by geriatricians and pharmacists within the research team (30–32).

**Table 2:** List of possible “alerts” for ADEs/ADWEs.

Type	Alert
Care alerts	Transfusion or use of blood products
	Code / arrest / rapid response team
	Acute dialysis / Acute kidney injury
	X-ray or Doppler studies for embolism or deep vein thrombosis
	Patient fall
	Immobilization
	In-hospital stroke
	Constipation
	Hypotension
	Lethargy
	Rash
	Unspecified adverse event
	Symptomatic bradycardia
	Delirium
Antidots and treatments	Vitamin K administration
	Antihistamines use
	Flumazenil use
	Naloxone use
	Antiemetic administration (for acute dyspepsia / nausea / vomiting)
	Haloperidol use
	Abrupt medication stop
Medication levels	Digoxin levels >2 ng/mL
	Carbamazepine levels >13 mcg/ml
Laboratory tests	Clostridioides difficile – positive stool
	Serum glucose <50 mg/dL
	Serum glucose >110 mg/dL
	INR > 5
	Haemoglobin or haematocrit decrease >25% / Acute bleeding
	Rising blood urea nitrogen or serum creatinine >2 times baseline
	Estimated glomerular filtration rate <35 mL/min/1.73 m2
	K > 6.0 mEq/L
	K < 2.9 mEq/L
	Na < 130 mEq/L
	ALT >80 U/L and/or AST > 84 U/L
	ALP > 350 U/L and/or total bilirubin >4 mg/dL
	CPK > 269 U/L
	TSH < 0.34 μUI/L or fT4 > 12 μg/dL
	White blood cells <3,000/μl
Emergency department	Emergency department visit within 48 h
	Time in emergency department >6 h
	Readmission within 30 days

In detail, we will evaluate alert diagnoses: unexpected clinical manifestations occurring during the admission and that might be related or unrelated to the use (or lack of use) of a certain medication. We will also evaluate previously defined altered laboratory tests, according to reference laboratory and literature review. Finally, we will evaluate alert drugs: medications known to be used to correct an alteration produced by a first medication (e.g., naloxone to reverse opioid overdose). All triggers will be further reviewed to discern if they are clearly independent from clinical manifestations related to the patients’ condition. Causality will be then assessed using the 10-point Naranjo Scale (33). The severity of the event will be classified using a modified Hartwig and Siegel scale (34), ranging from trivial (event does not require additional treatment and as no sequalae) to fatal. Avoidability of the event will be assessed using a modified Schumock and Thornton questionnaire (35). Each potential ADE/ADWE form will be evaluated by the blinded members of the ADE/ADWE Adjudication Committee, consisting of a geriatrician and a clinical pharmacist not directly involved in the retrospective review process. In the case of disagreement, additional review by another blinded geriatrician/clinical pharmacist pair of evaluators will be requested. Also, 90 days post-discharge (post-discharge assessment), both experimental and control phase participants will be re-evaluated for quantitative polypharmacy, PROMs, unplanned health care utilization (acute hospital admission, emergency claims), and mortality.

As for the qualitative part, semi-structured interviews will take place during index admission (for patients) and at the end of the study (for professionals). Interviews and focus groups will follow a topic guide and will be audio-recorded. Topic guide will focus on polypharmacy/treatment burden and interventions to improve drug adequacy. In detail, interviews and focus groups will follow a semi-structured guide and will be audio-recorded. We will explore participants’ experiences with polypharmacy and medication review, perceived barriers and facilitators to the implementation of the intervention, acceptability of the PCP approach, and suggestions for improving its integration into routine clinical practice. Recordings will be transcribed and analyzed using thematic analysis (36, 37). Two researchers will independently identify and refine themes through iterative discussion until consensus is reached. The qualitative findings will be used to contextualize and complement the quantitative findings, particularly with regard to implementation outcomes.

### Data analysis

Baseline variables will be described using means and standard deviations for quantitative variables and absolute and relative frequencies for categorical variables, with their corresponding 95% confidence intervals. The Student’s t-test will be used to compare means of medications and other qualitative variables, and the chi-squared test will be used to compare polypharmacy, hyperpolypharmacy, ADEs, ADWEs, and other categorical variables. All analyses will be stratified according to frailty and to age groups, as appropriate.

We will perform a generalized lineal model with mixed effects for (individual) time and cluster. In the descriptive analysis we also will perform descriptions for each cluster. The ICC for within-cluster correlation will be analyzed and recalculated with the data result and included in a sensitivity analysis.

Multivariable analyses will be carried out to assess the association between decrease in the number of medications at discharge and possible determinants. PROMs will be assessed by comparison of scores before and after the intervention by using paired t-test for continuous variables and McNemar’s test for categorical variables. Cox proportional hazard models will be used to evaluate the association between intervention and survival or time to first unplanned health care utilization. Missing data will be handled according to multivariate imputations by chained equations. Interim analyses are not planned.

All the analysis of secondary outcomes will only be exploratory as far as the sample size is not powered to detect differences on these variables.

All analyses will be carried out according to the intention-to-treat principle, with statistical significance at *p* < 0.05. STATA and R-Studio software will be used in their most recent versions available for statistical analysis.

## Discussion

The aim of the AMIDA-ICH trial is to evaluate the implementation and effectiveness of multidisciplinary PCP intervention for improving medication appropriateness in older adults to intermediate care hospitals. The idea of this project was conceived in the context of intermediate care, where patients with lower intensive care needs may benefit the most from a structured PCP. This is due to various reasons. First, the type of patients, often older, with a significant comorbidity burden, and a higher risk of adverse events because of their degree of frailty. As a result, they are also more likely to chronically take multiple medications, making them ideal candidates for PCP. Additionally, the reasons for their admission have often led to a worsening of the above-mentioned frailty status. However, PCP might also help improve frailty by aligning care with patients’ goals, as well as with medical, functional, and cognitive improvements. Furthermore, the involvement of a multidisciplinary team of specialists (including clinical pharmacists alongside the other “geriatric healthcare staff”) can, on the one hand, support frailty management, and, on the other, monitor potential complications that may arise during the process. This study is part of an established clinical and research framework in the centre where the research will take place, and this could contribute to the appropriateness and success of the project. The patients’ length of stay may also facilitate this process, ensuring the implementation of PCP.

A recent update of a Cochrane systematic review of 25 trials involving more than 15,000 patients, especially older ones, demonstrated that medication review likely reduces hospital admissions (38), and this is of particular importance, since it is known that the general population suffers from a significant deficit in therapeutic adherence and quality of care, posing a threat to public health (39). Focusing on intermediate care settings, it has been found that patients usually have a high prevalence of PIMs upon admission (40, 41), and it seems to be also associated with non-adherence to the pharmacological treatment (42). Nonetheless, it has also been demonstrated that intermediate care services may help reduce this inappropriateness (40, 41). Anyway, in order to derive a solution, a Spanish quasi-experimental study conducted on 93 patients, 81% of whom showing mild–moderate frailty, with an average age of 83 years, and a history of taking 5 or more medications for at least 3 months, showed that the PCP approach increased adherence, and reduced inappropriate prescriptions (21). The same group has provided additional evidence on the efficacy of PCP even in other settings, and the importance of tailoring treatments according to the patients’ goals (18, 19, 43). In spite of a solid methodology, all these studies are limited by relatively small sample sizes. Further evidence came from the D-PRESCRIBE cluster randomized trial, conducted in older community-dwelling people. It was found that a pharmacist-led intervention to patients and prescribers is more effective than the usual care in achieving deprescription (relative risk for discontinuation: 3.55) (44). Additionally, the INCREASE randomized-controlled trial, also involving older community-dwelling people, compared the usual recommendations on polypharmacy with an approach including a meeting with a pharmacist and a nurse or physician. It revealed that about 40% of “baseline” recommendations were revised following the meeting with specialists (45). Hence, these findings highlight the need for a strategy including different professionals, and the PCP itself is inherently complex and multidimensional. Additionally, since professionals alone might not suffice in the context of multidimensionality, the role of the patient should not be merely “passive.” This is why we decided to incorporate PROMs into the quantitative part of our complex intervention trial. To achieve our aims, we have designed a complex intervention study, including both a quantitative component, following the stepped-wedge trial design, and a qualitative part, with interviews and focus groups. There is not a universally accepted interpretation of complex interventions, but they generally include varying degrees of targets, flexibility, and interactions required (46). Therefore, the effectiveness of our intervention will surely largely depend on its implementation (47, 48), which is why we have chosen this design. Implementation is defined as the complex of strategies designed to support health interventions, with the aim of translating an intention into action (49–51), and we believe that, as mentioned, the AMIDA-ICH program fits within this scenario. Our aim is not only to evaluate the intervention’s efficacy in terms of “conventional” outcomes, such as a potential reduction of the number of medications per patient (our primary outcome), or even decreases in ADEs, but also to assess “implementation outcomes” (52). In particular, a significant role will be given to adherence, feasibility, and treatment burden, allowing us to evaluate the real-world applicability of our model, leveraging the perspectives of health professionals, patients, and caregivers.

We also acknowledge certain limitations and risks. First, the 90-day follow-up after discharge may vary among the patients, as some may stay at the ICH for longer or shorter periods, influencing the likelihood of following the appropriate therapeutic regimen. Also, we will not conduct specific interventions addressing the various geriatric domains (e.g., cognitive, affective, functional, nutritional), which would vary case by case and could likewise potentially confound the primary and secondary outcomes of the study, also considering the broad inclusion criteria. Moreover, as a monocentric study and the use of a reduced number of clusters, the reproducibility of our findings may be limited. The inclusion of additional centers could have strengthened the potential validity of the evidence deriving from the study. Additionally, given the multidisciplinary nature of the intervention, the applicability and reproducibility of the results in hospitals that are not specifically organized to provide comprehensive assessment and care of older people cannot be assumed and may be limited.

While our hospital maintains stable teams for each ward, we recognize that certain situations could lead to a risk of contamination. If the research team identifies a significant risk in this regard, recruitment may be withhold until the situation stabilizes.

Finally, regarding the sample size, statistical power was set to 75% for the primary outcome in order to guarantee viability of the project and we used previous data from a single center study. Thus, the results will have to be interpreted taking into consideration a possible overestimation of the effect.

Nonetheless, we also acknowledge several strengths. First, its mixed-model design, which is quite original in the literature. Additionally, the use of clusters may facilitate the implementation of the model. Furthermore, considering medications from both qualitative and quantitative perspectives, along with potential ADE/ADWEs, is crucial to enhancing the scientific value of the study, and providing more information on the model’s efficacy and safety.

Unlike previous studies (21, 28, 41, 44, 45), this trial combines a stepped-wedge cluster randomized design with an implementation science framework and an embedded qualitative evaluation. The intervention integrates multidisciplinary medication review, patient-reported outcome measures, and patients’ preferences within the intermediate care setting, providing evidence not only on effectiveness but also on implementation processes and contextual determinants.

To sum up, we believe that this study will offer a contribution to improving prescription appropriateness and reducing adverse events, particularly in the most vulnerable subjects, namely older, frail individuals with multiple comorbidities. By tailoring treatments to individual patient needs and preferences, we could enhance safety and efficacy of pharmacological interventions. Furthermore, as a future perspective, the implementation of this approach could strengthen the integration of pharmacological review strategies, ensuring a more comprehensive and safer management of medications.
