# Supplementary material for: Applying a multidisciplinary intervention for drug adequacy in an intermediate care hospital (AMIDA-ICH): study protocol of a complex intervention trial
Source: Front Med (Lausanne). 2026 Aug 19;13:1857969. doi: 10.3389/fmed.2026.1857969 (PMC13535294; doi:10.3389/fmed.2026.1857969)
Supplement: Supplementary file 1 [file Data_Sheet_1.pdf]

Hoja de Información al paciente para participar en un proyecto de investigación

(Este documento se firmará por duplicado quedándose una copia el investigador y otra el paciente**)**

**Título del estudio**	**Impacto de una intervención multidisciplinar basada en la adecuación farmacoterapéutica en pacientes ingresados en un hospital de Atención Intermedia**
**Código**	AMIDA-HAI
**Promotor**	La Fundació Hospital Universitari Vall d'Hebron-Institut de Recerca (VHIR)
**Investigador principal**	Ana María de Andrés Lázaro
**Centro**	Hospital Atención Intermedia Parc Sanitari Pere Virgili
**Servicio**	Farmacia
**Versión**	1, 12/04/2023

Introducción

Nos dirigimos a usted para informarle sobre un estudio de investigación en el que se le invita a participar. El estudio ha sido aprobado por un Comité de Ética de la Investigación con medicamentos.

Nuestra intención es que usted reciba la información correcta y suficiente para que pueda decidir si acepta o no participar en este estudio. Para ello lea esta hoja informativa con atención y nosotros le aclararemos las dudas que le puedan surgir.
Además, puede consultar con las personas que considere oportuno.

Participación voluntaria y derecho de revocación del consentimiento:

Debe saber que su participación en este estudio es voluntaria y que puede decidir no participar o cambiar su decisión y retirar el consentimiento en cualquier momento, sin que por ello se altere la relación con su médico ni se produzca perjuicio alguno en su tratamiento.

¿Por qué se le pide participar?

Usted ha sido invitado a participar en este estudio porque es un paciente que ha ingresado en nuestro centro de Atención Intermedia y recibe tratamiento con medicamentos (probablemente, más de 10 medicamentos diferentes).

Objetivos:
Le solicitamos su participación en este proyecto de investigación cuyo objetivo principal es obtener información sobre si el hecho de que un equipo de diferentes profesionales sanitarios (médicos, enfermeros y farmacéuticos) revisen los medicamentos que toma de manera habitual, puede conseguir reducir el número de fármacos que tiene que tomar (sin tener efecto perjudicial) a la vez que éstos sean más adecuados a sus características individuales y situación actual. Para ello se asignará al azar dos grupos de pacientes con el objetivo final de comparar la práctica clínica habitual con el nuevo método de revisión multidisciplinar para evaluar si hay diferencias en obtener un tratamiento individualizado, que sea más seguro a la vez que cumpla los objetivos personalizados para usted.

Beneficios:
Al tratarse de un estudio de investigación orientado a generar conocimiento no se espera que Vd. obtenga beneficio directo por participar, ni va a recibir ninguna compensación económica por ello, si bien usted contribuirá al avance del conocimiento y al beneficio social.

Procedimientos del estudio
Los investigadores recogerán datos referentes a su tratamiento y a sus características personales (su edad, función renal, movilidad, entre otras). No será necesario realizar ninguna prueba extraordinaria, y por supuesto, no se dejará de hacer ninguna prevista.
Si se le ha asignado al grupo de intervención (nuevo método de revisión), durante el ingreso le realizaremos diferentes cuestionarios para incluir sus preferencias sobre el tratamiento y le realizaremos un seguimiento adicional a los 3 meses del alta mediante consulta telefónica.
Cuando sea necesario, se informará de los resultados de la revisión a los profesionales que participan en la gestión de su medicación (personal de atención primaria, farmacia comunitaria).
Además, queremos realizar un seguimiento adicional posteriormente para evaluar la seguridad de su tratamiento.
Por otro lado, se desea conocer la opinión de los participantes (médicos, pacientes, cuidadores, etc) sobre esta manera de revisar la medicación. Por lo que, a alguno de los participantes, se le invitará a acudir a una reunión o entrevista (1 o 2 sesiones de 1-2 horas aproximadamente) en la que, mediante preguntas, podrá expresar su opinión. Para poder disponer de toda la información y realizar su análisis se grabará su voz y/o

imagen. Esta grabación únicamente se utilizará con finalidad de análisis durante la duración del estudio.

Molestias y posibles riesgos derivados de su participación en el estudio:
Se trata de un estudio en el que se evalúa la implementación de una nueva manera de trabajar en equipo, su tratamiento no va a cambiar por participar en este estudio. Si decide no participar recibirá la misma atención.

Protección de datos personales:

Tanto el promotor como el centro se asegurarán de que se mantenga la confidencialidad de sus datos personales recogidos durante el estudio, en cumplimiento tanto de las leyes de protección de datos nacionales como de las leyes europeas de protección de datos.

Para obtener más información sobre la confidencialidad y la protección de datos de carácter personal, consulte el Apéndice 1.

Contacto en caso de dudas
Si necesita más información sobre este estudio puede contactar con el investigador responsable, Ana María de Andrés del Servicio de **Farmacia***,* Tel. **93 259 4128**

Consentimiento informado

Título del estudio: AMIDA-HAI

Yo ___________________________

He leído la hoja de información que se me ha entregado.

He podido hacer preguntas sobre el estudio.

He hablado con: ___________________.

Comprendo que mi participación es voluntaria.

Comprendo que puedo retirarme del estudio:

1. Cuando quiera
2. Sin tener que dar explicaciones
3. Sin que esto repercuta en mis cuidados médicos

Presto libremente mi conformidad para participar en el estudio, confirmo que he leído el **Apéndice 1** y estoy conforme con su contenido.

Accedo a que los médicos responsables de este estudio me contacten en el futuro en caso de que se estime oportuno para añadir nuevos datos a los recogidos: Si__ No__

Firma del participante Firma del investigador

Fecha: ____/____/____ Fecha: ____/____/____

Firma del familiar/representante *(si procede)*

Fecha: ____/____/____

Deseo que me comuniquen la información derivada de la investigación que pueda ser relevante para mí salud o la de mis familiares: Si__ No__

Teléfono o e-mail de contacto: …………………….

Firma del participante Firma del investigador

Fecha: ____/____/____ Fecha: ____/____/____

firma del familiar/representante *(si procede)*

Fecha: ____/____/____

APARTADO PARA LA DENEGACIÓN O REVOCACION DEL CONSENTIMIENTO INFORMADO (FIRMA DEL PACIENTE Y/O FAMILIAR/REPRESENTANTE)

Yo, ...o el familiar/representante *(si procede)* ………………………………………………….. del paciente (nombre del paciente) ……………………………………………………………. deniego/revoco el consentimiento de participación en el estudio, arriba firmado.

Esta revocación de consentimiento informado supone que desde la fecha en la que se firma el mismo no se podrá recoger más datos médicos sin que esto perjudique a la conservación de los datos resultantes de las investigaciones que se hubiesen realizado con carácter previo.

Fecha de la denegación/revocación..

Firma:
